# Evaluation of methodologies to determine the effect of specific active immunotherapy on VEGF levels in phase I clinical trial patients with advanced solid tumors

**DOI:** 10.1016/j.heliyon.2018.e00906

**Published:** 2018-11-02

**Authors:** Javier Sánchez Ramírez, Mónica Bequet-Romero, Yanelys Morera Díaz, Francisco Hernández-Bernal, Ana de la Torre Santos, Katty-Hind Selman-Housein Bernal, Yenima Martín Bauta, Cimara H. Bermúdez Badell, Miladys Limonta Fernández, Marta Ayala Avila

**Affiliations:** a Department of Pharmaceuticals, Center for Genetic Engineering and Biotechnology, Playa, Havana, Cuba; b Department of Clinical Research, Center for Genetic Engineering and Biotechnology, Playa, Havana, Cuba; c “Celestino Hernández Robau” Hospital, Santa Clara, Villa Clara, Cuba; d Center of Medical and Surgical Research, Playa, Havana, Cuba

**Keywords:** Cancer research, Immunology

## Abstract

Two phase I clinical trials were conducted to evaluate, among other parameters, the humoral response elicited by a vascular endothelial growth factor (VEGF)-based therapeutic vaccine in cancer patients with advanced solid tumors. VEGF reduction was studied using an indirect methodology named as “Platelet VEGF”. This methodology is based on the estimation of VEGF within platelets by subtracting the plasma VEGF level from the serum level and dividing this by the platelet count, and then this latter expression is additionally corrected by the hematocrit. However, there is broad debate, whether serum or plasma VEGF or platelet-derived VEGF measurements is the most appropriate strategy to study the changes that occur on ligand bioavailability when patients are submitted to a VEGF-based immunotherapy.

The current research is a retrospective study evaluating the changes on VEGF levels in serum and plasma as well as platelet-derived measurements. Changes in VEGF levels were related with the humoral response seen in cancer patients after an active immunotherapy with a VEGF-based vaccine. The present study indicates that “Platelet VEGF” is the most reliable methodology to investigate the effect of VEGF-based immunotherapies on ligand bioavailability. “Platelet VEGF” was associated with those groups of individuals that exhibited the best specific humoral response and the variation of “Platelet VEGF” showed the strongest negative correlation with VEGF-specific IgG antibody levels. This methodology will be very useful for the investigation of this VEGF-based vaccine in phase II clinical trials and could be applied to immunotherapies directed to other growth factors that are actively sequestered by platelets.

## 1 Introduction

Pathological angiogenesis is one of the hallmarks of cancer [1]. Tumor-associated neovasculature, generated by the sprouting of new vessels from existing ones, is the source of nutrients and oxygen, indispensables for tumor expansion. Vascular endothelial growth factor (VEGF) is one of the most potent angiogenesis inducers, and is usually elevated in various types of tumor as well as in the circulation of cancer patients [2]. Therefore, VEGF or VEGF signaling pathway have become attractive targets for cancer immunotherapy [3].

With the increasing use of antiangiogenic agents for the treatment of cancer, either by blocking the ligand VEGF (ie: Bevacizumab) or by inhibiting the tyrosine kinase domain of VEGF receptors (ie: sorafenib, sunitinib), the measurements of VEGF have become an important tool for the assessment of *in vivo* efficacy of these agents or their combinations with cytostatic drugs [4, 5, 6]. Achieving predictability of therapeutic efficacy by measuring the decrease of the VEGF molecule is one the hardest goals to reach in passive or active immunotherapies directed to VEGF. For years, there has been much debate regarding whether serum or plasma is the best biological fluid to use for the measurement of VEGF. Some authors have preferably used serum samples [7] and others plasma instead of serum [8] because plasma VEGF levels have been considered a better assessment of any circulating VEGF released by the tumor [9, 10].

VEGF and other growth factors are actively sequestered by the platelets, and they are specifically stored inside secretory compartments: the α-granules [11]. Most recently, in addition to VEGF assessment in serum and plasma, platelet-derived VEGF measurements have been accepted, supported by clinical evidences of platelets scavenging of tumor-derived VEGF [12, 13] and pre-clinical data indicating that VEGF levels within platelets change significantly in the presence of the tumor, even in a tumor mass smaller than 1 mm^3^, which cannot be detected with conventional and applicable clinically methods [14]. Platelet-derived measurements include several approaches that estimate indirectly the VEGF content within the platelets. These approaches are based on assessment of serum VEGF normalized by the patient's platelet count [15], or by subtracting the plasma VEGF level from de serum level and dividing this by the platelet count [12], or this latter expression additionally corrected by the hematocrit [16].

In order to evaluate the value of all these strategies for VEGF measurement in the context of an active immunotherapy directed to this growth factor, this research work retrospectively investigated the changes in serum and plasma VEGF levels as well as platelet-derived measurements in two open and non-controlled phase I clinical trials, known as CENTAURO and CENTAURO-2 respectively [17, 18]. Both clinical trials were designed to study additionally the VEGF-specific humoral response elicited in patients with advanced solid tumors after active immunotherapy with a VEGF vaccine, known as CIGB-247. The antigen used is a recombinant fusion protein, representative of human VEGF isoform 121 [19] in combination with VSSP or aluminum phosphate as adjuvants. In this scenario, the present study analyzed which of the different approaches for VEGF measurements is more related to the specific humoral response seen in these vaccinated patients.

The results of this investigation indicate that VEGF content within the platelets is more relevant than either serum or plasma levels to study the changes on ligand availability after active immunization against human VEGF. Platelet VEGF as methodology will be applied to adequately powered efficacy trials of the VEGF-based vaccine.

## 2 Materials and methods

### 2.1 CENTAURO and CENTAURO-2 clinical trials

The present study analyzed 24 patients enrolled in CENTAURO clinical trial (phase Ia), as well as 38 individuals recruited in CENTAURO-2 clinical trial (phase Ib) [17, 18]. These two clinical studies, CENTAURO and CENTAURO-2, were conducted in accordance with the ethical guidelines of the Declaration of Helsinki. Written informed consent was obtained for all patients. Both clinical trials were approved by the hospitals institutional review boards and ethics committees (CIMEQ, Celestino Hernández Robau and José Ramón López Tabranes hospitals) and by the Cuban Regulatory Authority (CECMED).

Patients inclusion criteria were: histologically confirmed malignant solid tumors or metastases; measurable lesion(s); advanced disease that: (i) had shown to be refractory to available oncospecific therapies, was in progression, or was foreseen to rapidly progress and/or (ii) was not susceptible of further oncospecific treatment due to general patient status; off cancer therapy for ≥4 weeks; any sex and ages between 18 and 65 (both included); and Eastern Cooperative Oncology Group (ECOG) performance status ≤2.

Exclusion criteria included: patients with brain metastases, chronic un-compensated diseases, autoimmune or immune suppressing diseases or use of immune modulator drugs, moderate or severe systemic infections, individuals receiving biological therapies including active or passive immunotherapy, allergies to vaccine components, pregnancy or breast feeding, and evident mental incapacity to understand the trial information, deliver the consent, and act in consequence during the study [17, 18].

### 2.2 Cancer patients and vaccination groups

Patients recruited for both clinical trials had advanced tumors of a great variety of malignancies at original diagnosis that included breast, cervix, vulva, uterus, ovary, penis, lung, mediastinum, small intestine, colon, rectum, anal canal, gallbladder, kidney, soft-tissues, thyroid, pancreas, oropharynx and bone. Most individuals had disseminated disease [17, 18].

CENTAURO clinical trial studied three antigen levels with VSSP as adjuvant: (a) 50 μg of antigen + 200 μg of VSSP referred here in as ⅛ Ag + V; (b) 100 μg of antigen + 200 μg of VSSP referred here in as ¼ Ag + V; (c) 400 μg of antigen + 200 μg of VSSP referred here in as Ag + V. CENTAURO-2 study evaluated different antigen doses and adjuvants, taking as reference the highest antigen dose previously evaluated in the CENTAURO trial. The CENTAURO-2 study included the following groups: (d) 400 μg of antigen + 200 μg of VSSP referred here in as Ag + V; (e) 400 μg of antigen + 400 μg of VSSP referred here in as Ag+2V; (f) 800 μg of antigen + 200 μg of VSSP referred here in as 2Ag + V; (g) 200 μg of antigen + 0.7 mg of AlPO_4_ referred here in as ½Ag + Al; (h) 400 μg of antigen + 0.7 mg of AlPO_4_ referred here in as Ag + Al.

In VSSP-containing regimens, the trial vaccinations involved 8 weekly vaccinations, followed by a re-immunization on week 12. In aluminum phosphate-containing regimens, the trial vaccinations comprised 4 bi-weekly vaccinations, followed by a re-immunization on week 12.

Vaccine characteristics such as antigen lot number, adjuvants lot number, manufacturers, dosing interval and number of doses, and vaccine route administration have been previously described [17, 18]. CENTAURO clinical trial (RPCEC00000102) and CENTAURO-2 clinical trial (RPCEC00000155) are available from the Cuban Public Clinical Trial Registry at the URL http://registroclinico.sld.cu/.

### 2.3 Human blood samples

Venous blood samples were collected using a blood collection set with pre-attached holder (Becton Dickinson 367355) and taken into an EDTA tube (Becton Dickinson 367525) and into a serum separator tube (Greiner Bio-One 455092). Blood samples were centrifuged at 1800*g* for 10 minutes. The upper phase (serum or plasma) was transferred into a 2mL vial and immediately stored at -70 °C until use.

Blood samples from cancer patients were taken during the trial period at week 0 (pre-vaccination) and week 13 (one week after the end of trial vaccinations). Week 13 is one week after the ninth vaccination or the fifth vaccination in VSSP or aluminum-adjuvanted cohorts respectively [17, 18].

### 2.4 Measurements of VEGF

VEGF concentrations were measured with commercially available sandwich enzyme-linked immunosorbent assay kit from R&D Systems (catalogue No. SVE00). All standard reagents and solutions were used in accordance with the manufacturer's instructions. The minimum detectable dose of VEGF in this assay is 9.0 pg/mL, as quoted by the manufacturer. All samples were assayed in at least duplicated.

VEGF levels were measured in biological fluids: serum and plasma samples. Serum and plasma VEGF levels were expressed in picograms of VEGF per mL (pg/mL). The VEGF content within the platelets or platelet-derived measurements, expressed in picograms of VEGF/10^6^ platelet, was determined using several indirect methodologies, as has been previously described by other authors.

Platelet-corrected serum VEGF levels were calculated using the following formula [15]:

(1) $$platelet-corrected\;serum\;VEGF=\frac{serum\;VEGF}{platelet\;count}$$

Theoretical platelet-derived VEGF was calculated using the following formula [12]:

(2) $$platelet-derived\;VEGF=\frac{\left(serumVEGF-plasma\;VEGF\right)}{platelet\;count}$$

Hematocrit-corrected platelet VEGF was calculated using the following formula [16]:

(3) $$platelet\;VEGF=\frac{\left(serumVEGF-plasma\;VEGF\right)x\left(1-hematocrit\right)}{platelet\;count}$$

Platelet counts were performed on EDTA-anticoagulated blood samples and using an automated hematology analyzer (MINDRAY BC-3200).

The variation (Δ) of VEGF measurements induced by vaccination was expressed in percentage and was calculated using the following formula:

(4) $$\Delta =\left[\left(\frac{levels\;at\;week13}{levels\;at\;week0}\right)x100\right]-100\%$$

Based on criteria established by other authors [20], Δ ≤ -30% was considered a decrease; Δ ≥ 30% was considered an increase; -30% < Δ < 30% indicated a stability.

### 2.5 Statistical analysis

All statistical analyses were performed using GraphPad Prism software version 5.0 (La Jolla, CA). Parametric statistics were used for normally distributed data or after log conversion. Paired data were compared using the paired *t*-test. Non parametric statistics were used for data without normal distribution. In these cases, Mann-Whitney test was used for two-group comparisons and unpaired observations. Wilcoxon matched pairs test was used to evaluate differences of paired observations. Correlations between the variation of VEGF measurements and specific-VEGF IgG antibody titers (difference between antibody titer at week 13 and antibody titer at week 0) were evaluated using the Spearman correlation test. Statistical significance was considered as *p* < 0.05.

## 3 Results

### 3.1 VEGF baseline levels and hematological status of the patients recruited for CENTAURO and CENTAURO-2 clinical trials

**Fig. 1:**
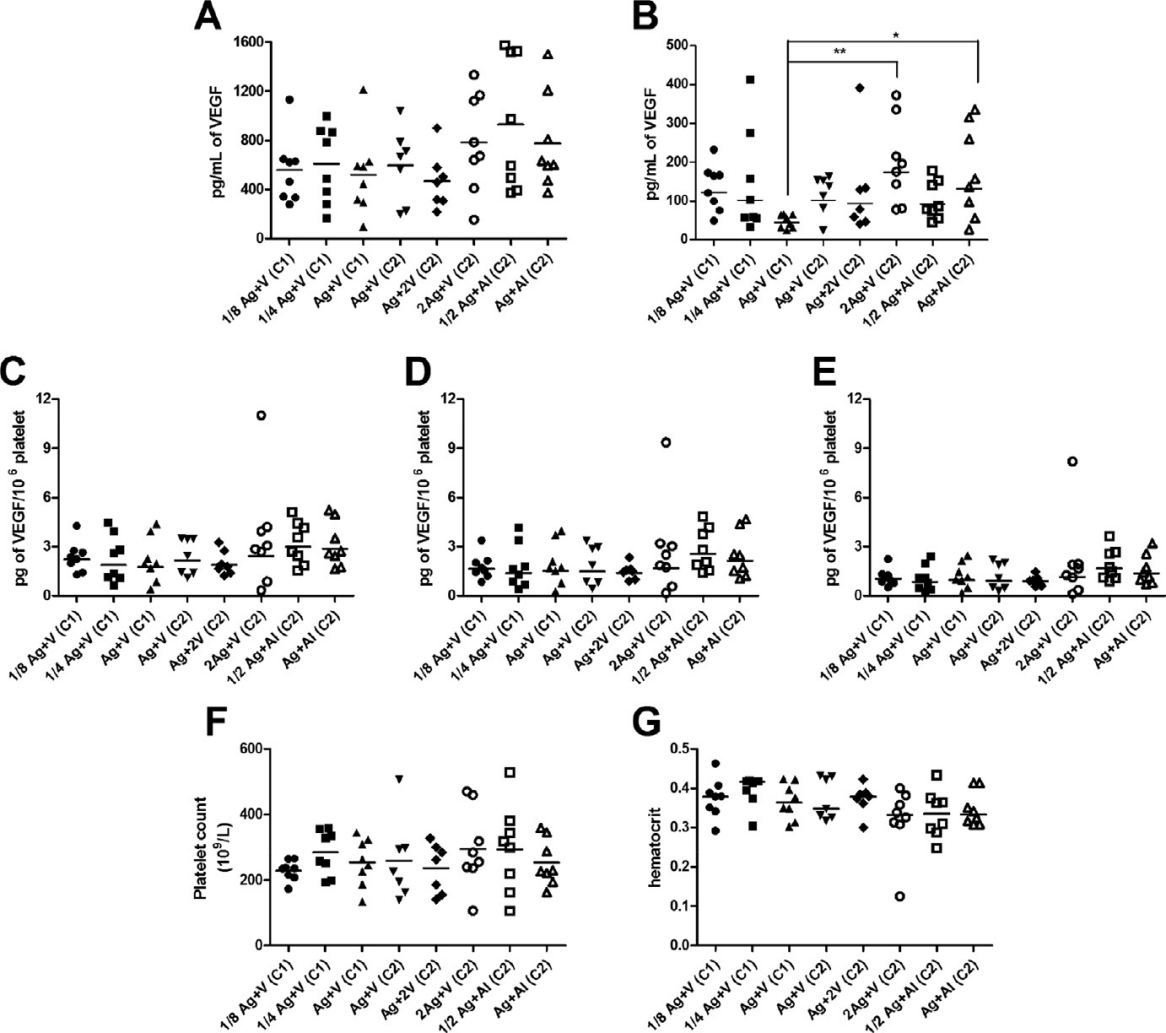
VEGF measurements and hematological values before initial vaccination (week 0). VEGF levels in biological fluids: in serum (A) and plasma (B). VEGF content within platelet: platelet-corrected serum VEGF (C); platelet-derived VEGF (D) and platelet VEGF (E). Hematological values: platelet count (F) and hematocrit (G). CENTAURO and CENTAURO-2 clinical trial were denoted as C1 and C2 respectively. Horizontal bars represent mean (A, F), geometric mean (B, C, D, E) or median (G). Statistical differences were calculated according one way ANOVA + Tukey's test or Kruskall-Wallis test + Dunn's test. (*) 0.01 < p < 0.05. (**) 0.001 < p < 0.01.

CENTAURO and CENTAURO-2 clinical trials comprise eight cohorts of patients, showing a great heterogeneity regarding the primary tumor types, the presence or not of metastasis and the ECOG status [17,18]. Serum and plasma VEGF levels as well as hematocrit values and platelet counts are parameters included in the different VEGF measurement approaches (serum VEGF, plasma VEGF, platelet-corrected serum VEGF [Eq. 1], platelet-derived VEGF [Eq. 2] or platelet VEGF [Eq. 3]) (Table S1). In order to determine the degree of heterogeneity between these groups of patients before initial vaccination, the values of these parameters at week 0 were compared (Fig. 1). No statistically significant differences were observed between the eight cohorts of patients in terms of serum VEGF (Fig. 1A), plasma VEGF (Fig. 1B), platelet counts (Fig. 1F) and hematocrit (Fig. 1G). Only the group Ag + V from CENTAURO clinical trial had VEGF plasma levels lower than the levels detected in the groups 2Ag + V and Ag + Al from CENTAURO-2 study (Fig. 1B). Neither there were any differences before initial vaccination in VEGF content within platelet: platelet-corrected serum VEGF (Fig. 1C); platelet-derived VEGF (Fig. 1D) and platelet VEGF (Fig. 1E). In general, all these results indicate that all groups were similar between them in VEGF baseline levels and hematological status in spite of their diverse background.

### 3.2 Measurements of VEGF levels in biological fluids and within platelets in cancer patients immunized with a VEGF-based vaccine

In order to investigate the dynamic changes on VEGF levels in patients immunized with a VEGF-based vaccine, two different compartments were studied: biological fluids (serum and plasma) and within platelets. Table 1 shows the results of VEGF measurements in patients recruited for the CENTAURO clinical trial. These patients were immunized with three different antigen dose levels, all in combination with a fixed quantity of VSSP as adjuvant. Two time points were evaluated: week 0 corresponding to the moment prior the initial vaccination, and week 13 corresponding to one week after the end of trial vaccinations.

**Table 1:** Measurements of VEGF prior and after active immunization with a VEGF-based vaccine in cancer patients enrolled in the CENTAURO clinical trial.

	VEGF measurements	Range Median [n]	
		Week 0	Week 13
**⅛ Ag + V**	**Serum VEGF**	279.4–1131 543.4 [8]	261.1–2312 579 [8] *ns*^∗∗^
	**Plasma VEGF**	49.5–232.1 142.4 [8]	33.3–484.6 120.5 [8] *ns*^∗∗^
	**platelet-corrected serum VEGF**	1.30–4.27 2.30 [8]	0.97–7.29 2.25 [8] *ns*^∗∗^
	**platelet-derived VEGF**	0.84–3.39 1.64 [8]	0.50–6.93 1.61 [8] *ns*^∗∗^
	**platelet VEGF**^a^	0.51–2.23 1.02 [8]	0.30–4.42 1.10 [8] *ns*^∗^
**¼ Ag + V**	**Serum VEGF**	168.9–1000 639.7 [8]	266.7–1971 414.0 [8] *ns*^∗∗^
	**Plasma VEGF**	34.0–412.2 81.15 [8]	45.9–816.5 145.3 [8] *ns*^∗∗^
	**platelet-corrected serum VEGF**	0.66–4.48 2.00 [8]	0.94–3.62 1.40 [8] *ns*^∗∗^
	**platelet-derived VEGF**	0.43–4.20 1.47 [8]	0.62–2.12 0.86 [8] *ns*^∗∗^
	**platelet VEGF**^a^	0.25–2.42 0.78 [8]	0.39–1.38 0.53 [8] *ns*^∗^
**Ag + V**	**Serum VEGF**	97.3–1213 514.1 [8]	133.9–1221 416.5 [8] *ns*^∗∗^
	**Plasma VEGF**	25.3–66.1 48.47 [8]	43.9–430.5 112.5 [8] p^**^=0.0040
	**platelet-corrected serum VEGF**	0.40–4.40 1.96 [8]	0.39–3.13 1.77 [8] *ns*^∗∗^
	**platelet-derived VEGF**	0.27–3.97 1.71 [8]	0.26–2.03 1.30 [8] *p*^∗∗^*=0.0264*
	**platelet VEGF**^a^	0.18–2.47 1.08 [8]	0.15–1.28 0.59 [8] *p*^∗^*=0.0156*

Legend: (n): number of evaluated patients. (^∗^): Wilcoxon matched pairs test were used for comparisons week 0 *vs.* week 13. (^∗∗^): paired *t*-test was used for comparisons week 0 *vs.* week 13. (ns): non-significant.

a Results that have been previously published [17]. Statistical significance was considered as *p* < 0.05.

In biological fluids (serum or plasma) a statistically significant increase on plasma VEGF levels with vaccination only occurred in the group Ag + V (*p* = 0.0040). The estimation of the VEGF content within the platelets using three different indirect methodologies showed that at week 13 a statistically significant reduction with vaccination only occurred in the group Ag + V in the cases of platelet-derived VEGF (*p* = 0.0264) and platelet VEGF (*p* = 0.0156).

CENTAURO-2 clinical trial was initiated to answer the question whether the immune response against VEGF, seen in patients from CENTAURO clinical trial could be increased, which could be further manipulated by increasing antigen dose and/or changing adjuvant composition in the CIGB-247 vaccine formulation [18]. Table 2 shows the results of VEGF measurements in patients recruited for the CENTAURO-2 clinical trial. These patients were immunized with different antigen doses and two distinct adjuvants: VSSP or aluminum phosphate.

**Table 2:** Measurements of VEGF prior and after active immunization with a VEGF-based vaccine in cancer patients enrolled in the CENTAURO-2 clinical trial.

	**VEGF measurements**	Range Mean [n]	
		Week 0	Week 13
**Ag + V**	**Serum VEGF**	199.9–1037 598.4 [7]	209–1274 466.5 [7] *ns*
	**Plasma VEGF**	24.6–163.5 118.3 [7]	62.6–422.8 191.6 [7] *ns*
	**platelet-corrected serum VEGF**	1.11–3.49 2.40 [7]	1.38–3.40 1.72 [7] *ns*
	**platelet-derived VEGF**	0.44–3.37 1.89 [7]	0.16–2.27 0.95 [7] *ns*
	**platelet VEGF**^a^	0.30–2.20 1.21 [7]	0.10–1.43 0.61 [7] *ns*
**Ag+2V**	**Serum VEGF**	218.4–901.2 469.9 [7]	56.0–2994 735.2 [7] *ns*
	**Plasma VEGF**	40.9–391.6 125.8 [7]	36.4–291.9 140.2 [7] *ns*
	**platelet-corrected serum VEGF**	1.22–3.27 2.00 [7]	0.36–5.28 2.08 [7] *ns*
	**platelet-derived VEGF**	0.90–2.35 1.48 [7]	0.13–4.77 1.56 [7] *ns*
	**platelet VEGF**^a^	0.55–1.46 0.93 [7]	0.08–3.29 1.01 [7] *ns*
**2Ag + V**	**Serum VEGF**	154.2–1333 785.3 [8]	180.9–1097 466.7 [8] *ns*
	**Plasma VEGF**	78.0–372.3 199.8 [8]	54.4–330.5 183.3 [8] *ns*
	**platelet-corrected serum VEGF**	0.34–11.02 3.62 [8]	0.49–3.34 1.44 [8] *p=0.0475*
	**platelet-derived VEGF**	0.17–9.36 2.80 [8]	0.29–2.95 0.85 [8] *p=0.0337*
	**platelet VEGF**^a^	0.11–8.19 2.07 [8]	0.22–2.05 0.57 [8] *p=0.0244*
**½Ag + Al**	**Serum VEGF**	373.9–1574 930.6 [8]	323.0–2553 1216.0 [8] *ns*
	**Plasma VEGF**	45.4–178.1 101.9 [8]	110.9–757.2 307.7 [8] *p=0.0264*
	**platelet-corrected serum VEGF**	1.56–5.10 3.22 [8]	1.45–6.98 3.94 [8] *ns*
	**platelet-derived VEGF**	1.42–4.85 2.82 [8]	0.95–5.45 2.93 [8] *ns*
	**platelet VEGF**^a^	0.90–3.64 1.90 [8]	0.63–3.54 1.98 [8] *ns*
**Ag + Al**	**Serum VEGF**	374.4–1499 774.0 [8]	86.9–1130 582.0 [8] *ns*
	**Plasma VEGF**	26.7–335.6 173.2 [8]	27.4–398.5 185.6 [8] *ns*
	**platelet-corrected serum VEGF**	1.66–5.22 3.11 [8]	0.39–4.84 2.38 [8] *p=0.0461*
	**platelet-derived VEGF**	1.05–4.68 2.45 [8]	0.27–4.22 1.66 [8] *p=0.0117*
	**platelet VEGF**^a^	0.72–3.19 1.58 [8]	0.15–2.91 1.06 [8] *p=0.0086*

Legend: (n): number of evaluated patients. (ns): non-significant.

a Results that have been previously published [18]. Paired *t*-test was used for comparisons week 0 *vs.* week 13. Statistical significance was considered as *p* < 0.05.

In biological fluids (serum or plasma) a statistically significant increase on plasma VEGF levels with vaccination only occurred in the group ½Ag + Al (*p* = 0.0264). The estimation of the VEGF content within the platelets using three different indirect methodologies (platelet-corrected serum VEGF, platelet-derived VEGF and platelet VEGF) showed that at week 13 a statistically significant reduction with vaccinations only occurred in the groups 2Ag + V and Ag + Al.

**Fig. 2:**
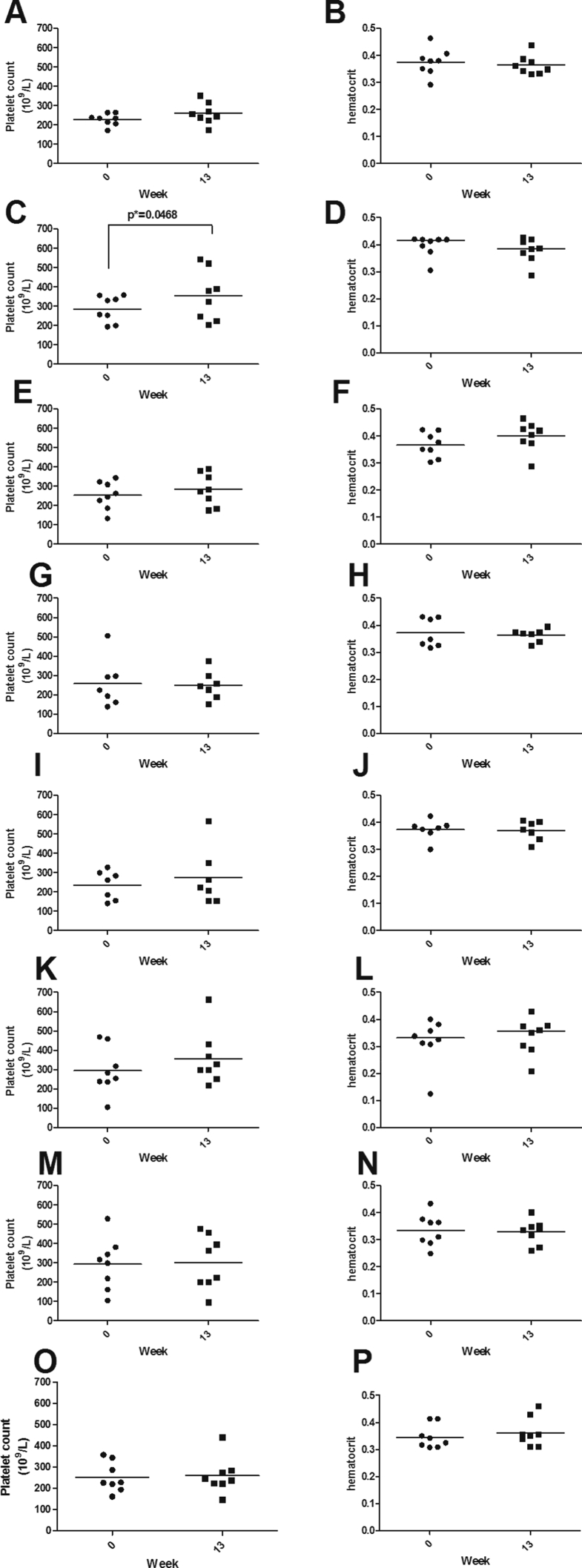
Comparisons of platelet count (A, C, E, G, I, K, M and O) and hematocrit (B, D, F, H, J, L, N and P) between weeks 0 and 13. From CENTAURO clinical trial: group ⅛ Ag + V (A and B); group ¼ Ag + V (C and D); group Ag + V (E and F). From CENTAURO-2 clinical trial: group Ag + V (G and H); group Ag+2V (I and J); group 2Ag + V (K and L); group ½Ag + Al (M and N); group Ag + Al (O and P). Horizontal bars represent mean (A, B, C, F, G, H, I, J, K, M, N and O), geometric mean (P) or median (D and L). Statistical differences were calculated according paired t-test or Wilcoxon matched pairs test. (*) 0.01 < p < 0.05.

Statistically significant variations were not observed between weeks 0 and 13 on platelet counts or hematocrit neither in CENTAURO clinical trial nor in CENTAURO-2 clinical study (Fig. 2). Only in the group ¼ Ag + V, platelet count values at week 13 were statistically significant higher than the values detected at week 0 (*p* = 0.0468) (Fig. 2C). All these results indicate that the changes observed in VEGF levels for some cohorts of patients from the two clinical trials could be associated with VEGF-targeted vaccinations and not to significant variations in platelet counts or hematocrit values.

### 3.3 Variations on VEGF measurements and VEGF specific-IgG antibody response

**Fig. 3:**
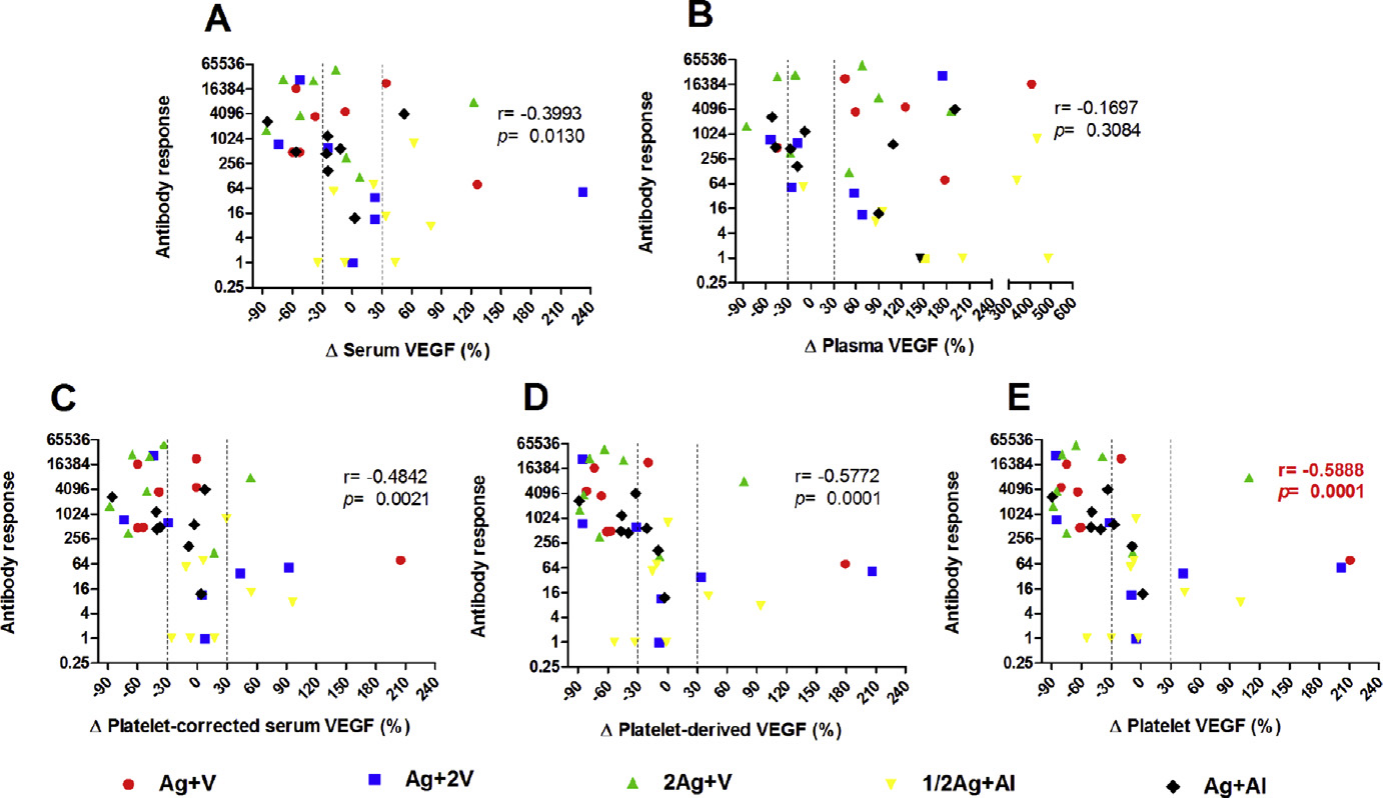
Correlation analysis between VEGF measurements and VEGF-specific IgG antibodies titers in CENTAURO-2 clinical trial. Results of non-parametric correlation at week 13. The variation (Δ) of the different VEGF measurements, expressed in percentages, was calculated as described in Materials and Methods: Δ Serum VEGF (A), Δ Plasma VEGF (B), Δ Platelet-corrected serum VEGF (C), Δ Platelet-derived VEGF (D) and Δ Platelet VEGF (E). Antibody response has been previously described [18] and represents VEGF-specific IgG antibody titers obtained from the difference between week 13 and week 0. Spearman r is Spearman correlation coefficient. Discontinued lines represent the cut-off values that indicate: ≥30% increase; ≤−30% decrease; between 30% and −30% stability. Statistical significance was considered as p < 0.05. Results that have been previously published are marked with bold in red text [18].

In general, the biological effects of both monoclonal and polyclonal antibodies are mediated by ligand depletion. CENTAURO-2 clinical trial was chosen in order to evaluate which of VEGF measurement approaches show the best association with the specific humoral response seen in cancer patients immunized with a VEGF-based vaccine. The patients from the CENTAURO study were excluded because the indirect ELISA to estimate serum levels of VEGF-specific IgG antibodies is less sensible than the ELISA test used for the CENTAURO-2 study [21]. As mentioned early, in CENTAURO-2 clinical trial, different antigen doses and adjuvants were evaluated. Irrespective of the used adjuvants or the antigen doses, the relationship between the variation of VEGF measurements (equation 4) and specific IgG antibodies titers (week 13 – week 0) was studied in 38 patients (Fig. 3).

As shown in Fig. 3, only Δ Plasma VEGF yielded no correlation with VEGF-specific IgG antibody titers (Fig. 3B). Δ Platelet-derived VEGF (Fig 3D) and Δ Platelet VEGF (Fig. 3E) showed the strongest negative correlation with antibody response (r = -0.5772 y r = -0.5888, respectively). In both VEGF measurements, the groups Ag + V, 2Ag + V and Ag + Al were the most representative in the area corresponding to high antibody titers associated with declines in VEGF levels within platelets (Figs. 3D and 3E).

After these results, it was interesting to investigate whether there was a match between those groups with an improved humoral response and those in which a significant decrease on values of VEGF measurements was observed.

Table 3 depicts a simple pooling of humoral response data detected in CENTAURO and CENTAURO-2 clinical trials [17, 18]. As shown in Table 3, in CENTAURO clinical trial the maximum dose (Ag + V) induced a higher humoral response. In most cases, this group occupied the first place in all the evaluated parameters (percentages of seroconversion, early IgG seroconversion and blocking activity) and the only one group where a statistically significant reduction in platelet-derived VEGF, platelet VEGF or a statistically significant increase in plasma VEGF levels was observed (Table 1). Although this group was the best of all, neither serum VEGF levels nor platelet-corrected serum VEGF did not experience any change associated with vaccinations (Table 1).

**Table 3:** Overall summary of humoral response results in CENTAURO and CENTAURO-2 clinical trials.

Parameters	CENTAURO clinical trial	CENTAURO-2 clinical trial
Percentages of patients with IgG seroconversion	Ag + V > ¼ Ag + V > ⅛ Ag + V	2Ag + V > Ag + V ≈ Ag + Al > Ag+2V > ½Ag + Al
Levels of VEGF-specific IgG antibodies at week 13	¼ Ag + V ≈ Ag + V > ⅛ Ag + V^∗^	2Ag + V > Ag + V > Ag + Al > Ag+2V > ½Ag + Al
Group with the highest percentages of patients with early IgG seroconversion	Ag + V	2Ag + V
Percentages of patients with blocking activity VEGF/VEGFR2	Ag + V > ¼ Ag + V > ⅛ Ag + V	2Ag + V ≈ Ag + V > Ag + Al ≈ ½Ag + Al > Ag+2V
Percentages of patients with blocking activity VEGF/VEGFR1	ND	2Ag + V ≈ Ag + Al > Ag+2V >½Ag + Al > Ag + V

Legend: (ND): not done. (*): these results are shown in Fig. 4.

In CENTAURO-2 clinical trial, of all VSSP-containing regimens, the group 2Ag + V exhibited the best humoral response, being the most relevant group for all evaluated parameters (Table 3). In this group there was a statistically significant decrease in platelet-corrected serum VEGF, platelet-derived VEGF and platelet VEGF (Table 2). Although this group was the best of all, neither serum VEGF levels nor plasma VEGF levels did not show a significant change associated with vaccinations. Similar results were obtained for the group of patients vaccinated with the highest antigen dose combined with aluminum phosphate (Ag + Al). Among aluminum phosphate-containing regimens, the group Ag + Al showed the best humoral response (Table 3) in which platelet-corrected serum VEGF, platelet-derived VEGF and platelet VEGF dropped significantly with vaccinations. Both, serum and plasma VEGF levels, did not change with vaccinations (Table 2).

**Fig. 4:**
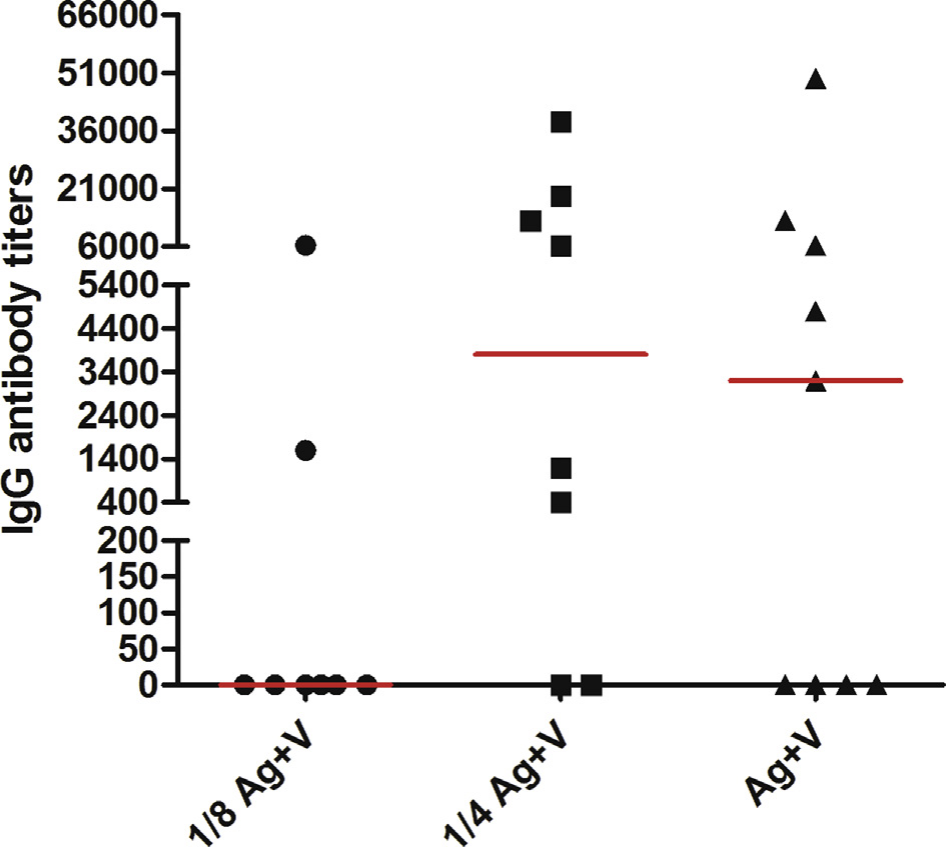
IgG specific antibody titers against human VEGF at week 13 in patients from CENTAURO clinical trial. Antibody titer at week 0 was subtracted from the antibody titer at week 13. Horizontal bars represent the median values of IgG antibody titers, which are shown for each group. (⅛ Ag + V): 50 μg of antigen + 200 μg of VSSP; (¼Ag + V): 100 μg of antigen + 200 μg of VSSP; (Ag + V): 400 μg of antigen + 200 μg of VSSP.

All results shown here are indicative that the specific humoral response elicited in patients by using a VEGF-based vaccine could be able to reduce *in vivo* VEGF bioavailability. The degree of this reduction is associated with those groups that exhibited the best humoral response induced by the optimal combination of antigen dose level and adjuvant. The relationship between the VEGF-specific antibody response and VEGF reduction is more relevant when VEGF measurements reflect the platelets inner compartment.

## 4 Discussion

The clinical success of VEGF-targeted immunotherapies like Bevacizumab in some tumors [22] has brought as result the need of the VEGF assessment with the aim to find out whether this growth factor could be depleted as result of this passive immunotherapy. During the extensive clinical evaluation of Bevacizumab, as monotherapy or concomitant with other anti-cancer treatments, VEGF measurements have been done in different biological fluids including serum or plasma [7, 8, 23] as well as other compartments such as platelets' interior [24]. Additionally, several works have indicated that the Bevacizumab effect on VEGF serum levels is opposite to the effect seen on plasma levels [4, 7].

CENTAURO clinical study was a first-in- human phase 1 clinical trial to evaluate a VEGF-based vaccine [17] and the question regarding where VEGF should be measured had not been still elucidated. Based on previously published works by other authors [2, 14, 16, 25], the methodology known as Platelet VEGF was chosen for VEGF measurements. Later, a second phase Ib clinical trial named as CENTAURO-2 was done to explore different antigen doses and two distinct adjuvants [18]. The same methodology for VEGF assessment was used in this case. Both clinical trials allowed us to increase the number of cancer patients undergoing immunization with a VEGF-based therapeutic vaccine. This fact led us to investigate, with a greater accuracy, whether Platelet VEGF established to evaluate the VEGF changes that occur as result of the specific humoral response induced by immunization was correct or whether VEGF assessments in serum or plasma were more relevant as has been previously described by other authors [26, 27, 28].

First of all, it is necessary to analyze the kit used for VEGF quantitation (R&D Systems, catalogue SVE00), one of the most widely used ELISA test for this purpose [2, 29]. It is based on an ELISA technique that employs a monoclonal antibody specific to VEGF as capture antibody, and an enzyme-linked polyclonal antibody specific for VEGF as detection antibody. If the polyclonal response specific to VEGF elicited in patients immunized with the vaccine binds to the same VEGF site that ELISA kit antibodies do (capture or detection), then a competition between antibodies occurs. An effective VEGF-specific polyclonal response could impair both the capture and/or the VEGF detection. Therefore, the VEGF quantitated by this kit in human serum or plasma samples from vaccinated patients could be considered as “free” VEGF. The detection of “free” VEGF by this kit has been previously demonstrated by Takahashi and colleagues for mixtures of VEGF with Bevacizumab [30]. In our case, the degree of VEGF detection decreases as the levels of elicited polyclonal antibodies is increased. This is in line with our findings, where an inverse and statistically significant correlation was observed between VEGF-specific IgG response and the variation of VEGF measurements.

However, some patients with high VEGF-specific IgG antibody titers did no show a decrease on platelet-associated VEGF. This finding could be explained by the differences in terms of quality of the resulting humoral response. In the two clinical studies, known as CENTAURO and CENTAURO-2, the percentages of patients with VEGF-blocking activity are lower than the percentages of patients with specific IgG antibodies [17, 18]. The specific polyclonal response developed by the vaccine candidate does not always achieve an effective blocking activity, and hence does not induce a decrease on VEGF bioavailability.

The other controversy is related to the fact that some patients with low VEGF-specific IgG antibody titers showed a decrease on platelet-associated VEGF. This decrease in VEGF levels could be attributed to the activation of specific T cell response, able to destroy VEGF-secreting tumor cells as well as tumor-associated stroma cells. Based on the scavenging of VEGF by platelets from the tumor source [12, 31], a reduction on the number of VEGF-secreting cells within tumor microenvironment could lead to decreased VEGF levels transported by platelets. Both preclinical and clinical evidences already published on this vaccine sustain that VEGF sources can also be depleted by cytotoxic T cells generated after vaccination. In mice C57Bl6 challenged with melanoma cells MB16-F10, the vaccine candidate using VSSP as adjuvant induced a cytotoxic T cell response with anti-tumoral activity, which could be mediated by CD8+ T lymphocytes. Vaccinated mice and treated with anti-CD8 antibodies significantly abrogated the anti-tumor effect as compared to the anti-tumor response observed in mice where no anti-CD8 treatment was done [19]. The induction of a VEGF-specific cellular response by the vaccine candidate has also been demonstrated in Balb/c mice [32], in non-human primates [33, 34] and using aluminum phosphate as alternative adjuvant [35]. Results from the CENTAURO clinical trial confirm this fact in tumor bearing human subjects that received the vaccine. Individuals negative for VEGF-specific IgG seroconversion had a positive test for the IFN-γ ELISPOT assay [17]. Furthermore, the evaluation of eight longer survivals subjects from the former trial indicated that the immune cell response could be mediated in part by VEGF-induced IFN- γ secreting-CD8+ cells [36]. This evidence is indicative that this therapeutic vaccine can stimulate the production of VEGF-specific cytotoxic CD8+ cells, the latter with a potential role on the vaccine's anti-tumor mechanisms.

Both types of behaviors (high antibody titer with no decrease on VEGF and low antibody titer with decrease on VEGF) only occurred in a relatively small number of patients, hence this fact did not affect the conclusions of the statistical tests accounting for significant negative correlation between platelet-associated VEGF and the IgG antibody response.

Within this context, it must be expected that groups of patients with an improved humoral response against VEGF match with those groups in which a significant decrease on values of VEGF measurements was observed. Additionally, as has been commented in the previous paragraph, the most informative VEGF measurement should be one that shows a higher inverse correlation with the antibody response. Both assumptions were used to decide which of the VEGF measurements, in biological fluids (serum or plasma) or the VEGF content within the platelets, are more relevant to study the changes in this growth factor after patients immunization with a VEGF-based vaccine.

Plasma sample is the less recommended for VEGF measurements in patients treated with a VEGF vaccine. A significant decrease in plasma VEGF levels was not associated to the group of patients that exhibited the best humoral response, neither in the CENTAURO study nor in the CENTAURO-2 study. In addition, there was no correlation between the variation of plasma VEGF and VEGF-specific IgG antibody response. The presence of VEGF in plasma samples as a consequence of *ex vivo* platelet activation during the blood harvest procedure has been suggested by other authors [25].

For serum sample, a significant inverse correlation was observed between the variation of serum VEGF and VEGF-specific IgG antibody response. A similar result was found by other authors in a phase III study of an EGF-based vaccine for the treatment of patients with non-small cell lung cancer. These authors observed the same association between the anti-EGF antibody titers and serum EGF concentration [37]. Although a relationship was found between both parameters (variation of serum VEGF levels *vs* specific antibody response), of all significant correlations, this one was the weakest. This fact could explain why there was no change on serum VEGF levels neither in the CENTAURO study nor in the CENTAURO-2 study for those groups of patients that exhibited the best humoral response.

Three different approaches to estimate the VEGF content within the platelets have been previously published: Platelet-corrected serum VEGF levels [15], Platelet-derived VEGF [12] and Platelet VEGF [16]. In the present study, the variation of all these measurements was correlated with the VEGF-specific IgG antibody response. Platelet-derived VEGF or Platelet VEGF values showed a significant decrease in the group of patients that exhibited the best humoral response, specifically the group Ag + V from CENTAURO study, and in CENTAURO-2 clinical trial, the groups 2Ag + V (the best group of all VSSP-containing regimens) and Ag + Al (the best group of aluminum phosphate-containing regimens). However, a different result was observed for Platelet-corrected serum VEGF. The values obtained using this methodology did not change in the group Ag + V, which showed the best humoral response detected in CENTAURO clinical trial. This result can be explained by the fact that of all VEGF measurements within platelets, this type of methodology had the weakest negative correlation with antibody response.

Based on the results of this investigation and preceding works published by other authors, the use of “Platelet VEGF” is more appropriate to study the changes that occur on VEGF bioavailability under a VEGF-based immunotherapy: (a) the variation of “Platelet VEGF” showed the strongest negative correlation with specific antibody response; (b) a significant decrease on “Platelet VEGF” values was associated with those groups of patients that exhibited the best humoral response in CENTAURO and CENTAURO-2 clinical trials; (c) Hematological parameter such as platelet counts, the major physiological transporter of VEGF in blood, can influence on serum VEGF levels in cancer patients [12, 13, 16]. The intersubject biovariability or the variation over time in the same individual, in terms of platelet counts and the packet cell volume (hematocrit) are corrected using this methodology.

This work was retrospectively tried, and involved the evaluation of a VEGF-based vaccine in two phase I clinical trials, but there are some weak points that deserve to be pointed out. First of all, our sample is limited in size, either the overall sample or the number of patients evaluated per group, as well as the aspect that patients had different types of malignancies at original diagnosis [17, 18]. All these aspects explain the following fact: In the CENTAURO study, the group Ag + V showed statistically significant reduced levels of “Platelet-derived VEGF” or “Platelet VEGF” with respect to pre-vaccination values, however, in the CENTAURO-2 clinical trial, this drop in VEGF was not observed in the group with the same dose and schedule [17, 18]. Despite this limitation, the finding of VEGF variations in the context of an active immunotherapy in patients with different tumors is of great importance. Accumulating this type of data through this retrospective study about the evaluation of a VEGF vaccine in humans can provide the necessary support for establish a better way of VEGF measuring. For example, the experiences obtained from these two phase I clinical trials will be very useful for the investigation of this VEGF-based vaccine in phase II clinical trials, where a larger number of patients with the same type of tumor will be included (Trial registration numbers: RPCEC00000237 and RPCEC00000246). Therefore, the variation of Platelet VEGF, the best of all VEGF measurements evaluated in this work, could provide the basis for the establishment of a correlation between this VEGF measurement and clinical outcome after immunization with the vaccine candidate.

Secondly, the measurement of “Platelet VEGF” is an indirect methodology that estimates the VEGF content within the platelets. This approach assumes that during the blood clotting process, platelets release all its VEGF content upon activation, and hence, serum measurement include all VEGF found in platelets [12]. However, some VEGF levels remain associated with the activated platelets [14]. To overcome this problem, serum separator tubes containing a serum clot activator were used in order to induce the maximum platelet activation. Other authors have described direct measurements of the concentration of VEGF within the platelets, in which the platelets are isolated, lysed and then the extract is analyzed for VEGF content [24, 38, 39]. However, with the use of this strategy, it is necessary the standardization of an ELISA to quantify the number of platelets in the sample [38].

Several studies have focused on VEGF measurements within the platelets because of three principal reasons: (a) platelets are considered the major physiological transporter of VEGF in blood [2, 40]; (b) peripheral blood platelets of cancer patients carry more VEGF than platelets of normal controls [12, 39, 41]; (c) platelets are actively infiltrated to solid tumors and have a relevant role in tumor angiogenesis [42, 43, 44]. For example, this study retrospectively investigated the changes that occur on VEGF bioavailability inside the platelets of cancer patients treated with a VEGF-based vaccine. However, other blood cell components including natural killer (NK) cells and neutrophils are not studied. Similar to platelets, peripheral NK cells and neutrophils of cancer patients produce or transport more VEGF than that of healthy controls [45, 46, 47]. Tumors can polarize both blood cells to a pro-angiogenic phenotype, which could contribute to tumor progression [48, 49]. The effect of this VEGF-based active immunotherapy on VEGF levels within these blood cell components could be analyzed in future investigations.

Platelets are actively infiltrated to solid tumors [42], serving as a source of VEGF, a pro-angiogenic factor indispensable for survival, proliferation and migration of the tumor associated-endothelial cells [50, 51]. Therefore, VEGF secreted by platelets is considered one of the key factors that promotes tumor angiogenesis [52]. In the present work, there is evidence that active immunization directed to this growth factor is able to reduce *in vivo* the levels of platelet associated-VEGF. In this sense, it is possible to consider VEGF vaccination as an alternative strategy for the impairment of tumor angiogenesis. VEGF has also immunosuppressive properties, inhibiting the biological activity of immune cells such as dendritic cells and cytotoxic CD8 T cells, both with important role in tumor cells elimination, or activating the regulatory CD4 T cells and myeloid-derived suppressor cells that support the local immunosuppression within tumor microenvironment [53, 54]. A reduction of tumor VEGF induced by the vaccine candidate could lead to a restoration of anti-tumor immunity in vaccinated cancer patients.

To end the discussion, it is necessary to point out about the possible importance of this work. Being this vaccine candidate the first VEGF-specific active immunotherapy evaluated in humans, to our knowledge, this is the first report that analyzes which of the VEGF measurements, in biological fluids or within the platelets, is most appropriate to study the changes that occur on VEGF bioavailability under a VEGF-based active immunotherapy. Similar to VEGF, there are other growth factors actively sequestered by platelets such as platelet-derived growth factor, fibroblast growth factor or epidermal growth factor [11, 38, 41, 55]. Consequently, clinical trials of immunotherapies targeting these growth factors could benefit from the experiences included in the present study.

## 5 Conclusions

In this study, platelet–derived measurements are more relevant than the VEGF levels in biological fluids such as serum and plasma. Among platelet-derived measurements, Platelet VEGF is the most reliable methodology to evaluate the dynamic changes that occur on VEGF levels in patients submitted to active immunotherapies directed to this growth factor. All these experiences could be applied in future clinical trials of the vaccine or in other vaccines directed to growth factors sequestered by the platelets.

## Declarations

### Author contribution statement

Javier Sánchez Ramírez: Conceived and designed the experiments; performed the experiments; Analyzed and interpreted the data; Wrote the paper.

Mónica Bequet Romero, Yanelys Morera Díaz: Conceived and designed the experiments; Analyzed and interpreted the data; Wrote the paper.

Francisco Hernández Bernal, Ana de la Torre Santos, Katty-Hind Selman-Housein Bernal, Yenima Martín Bauta, Cimara H. Bermúdez Badell, Miladys Limonta Fernández: Analyzed and interpreted the data; Contributed reagents, materials, analysis tools or data.

Marta Ayala Avila: Conceived and designed the experiments; Analyzed and interpreted the data.

### Funding statement

This work was supported by Heber Biotec and the Ministry of Public Health of Cuba. They were involved in all stages of the study conduct and analysis.

### Competing interest statement

The authors declare the following conflict of interests: Mónica Bequet-Romero is the inventor on a patent application submitted by the Center for Genetic Engineering and Biotechnology that covers the use of the vaccine. This fact did not alter adherence to Heliyon policies on sharing data and materials.

### Additional information

The clinical trials described in this paper are registered at the Cuban Public Clinical Trial Registry under the registration numbers RPCEC00000102 and RPCEC00000155.
