# Supplementary material for: Evaluation of methodologies to determine the effect of specific active immunotherapy on VEGF levels in phase I clinical trial patients with advanced solid tumors
Source: Heliyon. 2018 Nov 2;4(11):e00906. doi: 10.1016/j.heliyon.2018.e00906 (PMC6223189; doi:10.1016/j.heliyon.2018.e00906)
Supplement: Table S1 [file mmc1.docx]

**Table S1. Measurements of VEGF and hematological status of the patients recruited for CENTAURO and CENTAURO-2 clinical trials.**

|  | **CENTAURO** | |
| --- | --- | --- |
|  | **Group ⅛Ag+V** | |
|  | **Serum VEGF (pg/mL)** | |
|  | **Week 0** | **Week 13** |
| CH01 | 629.79 | 266.76 |
| CH02 | 622.97 | 775.30 |
| CH03 | 334.22 | 596.36 |
| CH06 | 342.28 | 561.69 |
| CH07 | 279.39 | 261.60 |
| CH08 | 463.74 | 261.09 |
| CH09 | 1131.42 | 2311.81 |
| CH10 | 646.86 | 851.32 |

|  | **CENTAURO** | |
| --- | --- | --- |
|  | **Group ¼Ag+V** | |
|  | **Serum VEGF (pg/mL)** | |
|  | **Week 0** | **Week 13** |
| CH11 | 387.39 | 355.95 |
| CH14 | 168.88 | 357.07 |
| CH15 | 868.73 | 399.63 |
| CH16 | 878.64 | 642.54 |
| CQ17 | 784.94 | 428.41 |
| CH25 | 284.55 | 266.66 |
| CH26 | 1000.07 | 526.03 |
| CH27 | 494.37 | 1970.71 |

|  | **CENTAURO** | |
| --- | --- | --- |
|  | **Group Ag+V** | |
|  | **Serum VEGF (pg/mL)** | |
|  | **Week 0** | **Week 13** |
| CH18 | 583.96 | 419.01 |
| CH19 | 97.28 | 268.52 |
| CH21 | 1213.34 | 1220.71 |
| CH22 | 444.26 | 414.05 |
| CH23 | 591.90 | 620.50 |
| CH24 | 627.52 | 519.31 |
| CH29 | 293.43 | 133.94 |
| CH30 | 317.49 | 296.29 |

|  | **CENTAURO-2** | |
| --- | --- | --- |
|  | **Group Ag+V** | |
|  | **Serum VEGF (pg/mL)** | |
|  | **Week 0** | **Week 13** |
| CQ03 | 668.48 | 314.14 |
| CH04 | 563.62 | 1273.77 |
| CH17 | 711.98 | 446.67 |
| CH24 | 224.86 | 209.02 |
| CH32 | 783.08 | 341.10 |
| JL41 | 199.64 | 267.72 |
| JL42 | 1037.44 | 413.10 |

|  | **CENTAURO-2** | |
| --- | --- | --- |
|  | **Group Ag+2V** | |
|  | **Serum VEGF (pg/mL)** | |
|  | **Week 0** | **Week 13** |
| CH15 | 319.52 | 392.76 |
| CH19 | 308.34 | 144.51 |
| CH27 | 460.31 | 347.17 |
| CH39 | 577.74 | 707.13 |
| JL49 | 218.44 | 56.04 |
| JL22 | 504.07 | 504.81 |
| CQ44 | 901.16 | 2993.78 |

|  | **CENTAURO-2** | |
| --- | --- | --- |
|  | **Group 2Ag+V** | |
|  | **Serum VEGF (pg/mL)** | |
|  | **Week 0** | **Week 13** |
| CH16 | 640.46 | 533.71 |
| JL29 | 409.90 | 441.27 |
| JL30 | 1167.81 | 1096.53 |
| CH33 | 1121.35 | 341.73 |
| JL43 | 154.15 | 342.93 |
| CH50 | 1333.21 | 180.87 |
| JL12 | 672.57 | 319.68 |
| CH35 | 782.75 | 476.81 |

|  | **CENTAURO-2** | |
| --- | --- | --- |
|  | **Group ½Ag+Al** | |
|  | **Serum VEGF (pg/mL)** | |
|  | **Week 0** | **Week 13** |
| CH08 | 391.80 | 363.07 |
| CH09 | 373.88 | 669.73 |
| CH20 | 1519.81 | 1846.30 |
| CH25 | 494.32 | 323.04 |
| CH46 | 592.71 | 482.45 |
| JL47 | 974.32 | 1301.51 |
| CH18 | 1524.20 | 2185.16 |
| JL23 | 1574.00 | 2552.94 |

|  | **CENTAURO-2** | |
| --- | --- | --- |
|  | **Group Ag+Al** | |
|  | **Serum VEGF (pg/mL)** | |
|  | **Week 0** | **Week 13** |
| CH07 | 593.69 | 439.21 |
| CQ13 | 807.44 | 711.95 |
| CQ28 | 472.73 | 719.89 |
| CH37 | 635.49 | 275.34 |
| CH45 | 600.81 | 86.93 |
| JL48 | 1499.09 | 1130.20 |
| JL11 | 374.37 | 383.39 |
| CH10 | 1208.77 | 909.47 |

|  | **CENTAURO** | |
| --- | --- | --- |
|  | **Group ⅛Ag+V** | |
|  | **Plasma VEGF (pg/mL)** | |
|  | **Week 0** | **Week 13** |
| CH01 | 120.44 | 124.23 |
| CH02 | 166.92 | 144.60 |
| CH03 | 49.54 | 36.16 |
| CH06 | 76.13 | 66.01 |
| CH07 | 99.71 | 33.25 |
| CH08 | 164.42 | 127.06 |
| CH09 | 232.12 | 116.69 |
| CH10 | 173.23 | 484.60 |

|  | **CENTAURO** | |
| --- | --- | --- |
|  | **Group ¼Ag+V** | |
|  | **Plasma VEGF (pg/mL)** | |
|  | **Week 0** | **Week 13** |
| CH11 | 159.53 | 153.58 |
| CH14 | 57.27 | 45.86 |
| CH15 | 56.42 | 186.78 |
| CH16 | 276.50 | 402.04 |
| CQ17 | 102.97 | 137.07 |
| CH25 | 59.33 | 48.18 |
| CH26 | 412.22 | 78.88 |
| CH27 | 33.99 | 816.45 |

|  | **CENTAURO** | |
| --- | --- | --- |
|  | **Group Ag+V** | |
|  | **Plasma VEGF (pg/mL)** | |
|  | **Week 0** | **Week 13** |
| CH18 | 56.06 | 69.44 |
| CH19 | 31.47 | 177.74 |
| CH21 | 65.31 | 430.46 |
| CH22 | 63.81 | 130.47 |
| CH23 | 40.88 | 108.85 |
| CH24 | 66.06 | 116.08 |
| CH29 | 25.31 | 43.85 |
| CH30 | 32.07 | 55.14 |

|  | **CENTAURO-2** | |
| --- | --- | --- |
|  | **Group Ag+V** | |
|  | **Plasma VEGF (pg/mL)** | |
|  | **Week 0** | **Week 13** |
| CQ03 | 113.46 | 62.58 |
| CH04 | 152.24 | 422.77 |
| CH17 | 163.49 | 260.10 |
| CH24 | 81.83 | 184.50 |
| CH32 | 24.55 | 124.67 |
| JL41 | 138.86 | 201.56 |
| JL42 | 153.46 | 84.87 |

|  | **CENTAURO-2** | |
| --- | --- | --- |
|  | **Group Ag+2V** | |
|  | **Plasma VEGF (pg/mL)** | |
|  | **Week 0** | **Week 13** |
| CH15 | 59.44 | 93.08 |
| CH19 | 40.86 | 112.20 |
| CH27 | 129.41 | 106.51 |
| CH39 | 133.65 | 223.93 |
| JL49 | 78.79 | 36.37 |
| JL22 | 46.84 | 117.56 |
| CQ44 | 391.57 | 291.94 |

|  | **CENTAURO-2** | |
| --- | --- | --- |
|  | **Group 2Ag+V** | |
|  | **Plasma VEGF (pg/mL)** | |
|  | **Week 0** | **Week 13** |
| CH16 | 196.65 | 330.49 |
| JL29 | 143.87 | 216.76 |
| JL30 | 175.78 | 128.38 |
| CH33 | 215.48 | 170.87 |
| JL43 | 77.98 | 147.89 |
| CH50 | 372.31 | 54.43 |
| JL12 | 80.88 | 231.31 |
| CH35 | 335.54 | 186.34 |

|  | **CENTAURO-2** | |
| --- | --- | --- |
|  | **Group ½Ag+Al** | |
|  | **Plasma VEGF (pg/mL)** | |
|  | **Week 0** | **Week 13** |
| CH08 | 55.90 | 168.45 |
| CH09 | 78.64 | 146.25 |
| CH20 | 86.68 | 380.30 |
| CH25 | 45.36 | 110.94 |
| CH46 | 178.11 | 160.21 |
| JL47 | 153.01 | 297.24 |
| CH18 | 75.48 | 441.29 |
| JL23 | 142.04 | 757.15 |

|  | **CENTAURO-2** | |
| --- | --- | --- |
|  | **Group Ag+Al** | |
|  | **Plasma VEGF (pg/mL)** | |
|  | **Week 0** | **Week 13** |
| CH07 | 315.68 | 229.83 |
| CQ13 | 97.51 | 203.67 |
| CQ28 | 136.97 | 398.47 |
| CH37 | 260.02 | 137.83 |
| CH45 | 56.00 | 27.36 |
| JL48 | 157.12 | 129.19 |
| JL11 | 26.68 | 50.71 |
| CH10 | 335.59 | 308.11 |

|  | **CENTAURO** | |
| --- | --- | --- |
|  | **Group ⅛Ag+V** | |
| **Platelet-corrected serum VEGF (pg/10^6^)** | | |
|  | **Week 0** | **Week 13** |
| CH01 | 2.646 | 1.116 |
| CH02 | 2.360 | 2.203 |
| CH03 | 1.428 | 3.408 |
| CH06 | 1.990 | 2.302 |
| CH07 | 1.299 | 1.163 |
| CH08 | 2.240 | 0.967 |
| CH09 | 4.270 | 7.293 |
| CH10 | 2.753 | 3.313 |

|  | **CENTAURO** | |
| --- | --- | --- |
|  | **Group ¼Ag+V** | |
| **Platelet-corrected serum VEGF (pg/10^6^)** | | |
|  | **Week 0** | **Week 13** |
| CH11 | 1.177 | 1.102 |
| CH14 | 0.657 | 0.937 |
| CH15 | 4.478 | 1.625 |
| CH16 | 2.623 | 1.652 |
| CQ17 | 3.944 | 2.100 |
| CH25 | 1.129 | 1.196 |
| CH26 | 2.809 | 1.010 |
| CH27 | 1.381 | 3.623 |

|  | **CENTAURO** | |
| --- | --- | --- |
|  | **Group Ag+V** | |
| **Platelet-corrected serum VEGF (pg/10^6^)** | | |
|  | **Week 0** | **Week 13** |
| CH18 | 4.391 | 2.381 |
| CH19 | 0.399 | 1.128 |
| CH21 | 3.927 | 3.130 |
| CH22 | 1.966 | 2.263 |
| CH23 | 2.251 | 2.177 |
| CH24 | 1.943 | 1.370 |
| CH29 | 0.853 | 0.387 |
| CH30 | 1.698 | 1.077 |

|  | **CENTAURO-2** | |
| --- | --- | --- |
|  | **Group Ag+V** | |
| **Platelet-corrected serum VEGF (pg/10^6^)** | | |
|  | **Week 0** | **Week 13** |
| CQ03 | 3.446 | 1.384 |
| CH04 | 1.114 | 3.397 |
| CH17 | 2.430 | 1.494 |
| CH24 | 1.388 | 1.375 |
| CH32 | 3.480 | 1.392 |
| JL41 | 1.436 | 1.424 |
| JL42 | 3.493 | 1.595 |

|  | **CENTAURO-2** | |
| --- | --- | --- |
|  | **Group Ag+2V** | |
| **Platelet-corrected serum VEGF (pg/10^6^)** | | |
|  | **Week 0** | **Week 13** |
| CH15 | 1.224 | 1.746 |
| CH19 | 1.667 | 0.932 |
| CH27 | 3.265 | 2.314 |
| CH39 | 1.932 | 2.020 |
| JL49 | 1.409 | 0.364 |
| JL22 | 1.775 | 1.912 |
| CQ44 | 2.756 | 5.280 |

|  | **CENTAURO-2** | |
| --- | --- | --- |
|  | **Group 2Ag+V** | |
| **Platelet-corrected serum VEGF (pg/10^6^)** | | |
|  | **Week 0** | **Week 13** |
| CH16 | 2.680 | 1.773 |
| JL29 | 0.872 | 1.021 |
| JL30 | 11.017 | 3.343 |
| CH33 | 3.948 | 1.361 |
| JL43 | 0.336 | 0.517 |
| CH50 | 4.192 | 0.493 |
| JL12 | 2.850 | 1.402 |
| CH35 | 3.070 | 1.600 |

|  | **CENTAURO-2** | |
| --- | --- | --- |
|  | **Group ½Ag+Al** | |
| **Platelet-corrected serum VEGF (pg/10^6^)** | | |
|  | **Week 0** | **Week 13** |
| CH08 | 2.434 | 1.806 |
| CH09 | 3.561 | 6.976 |
| CH20 | 4.418 | 4.674 |
| CH25 | 1.559 | 1.449 |
| CH46 | 2.706 | 2.400 |
| JL47 | 1.845 | 2.848 |
| CH18 | 5.098 | 5.987 |
| JL23 | 4.131 | 5.352 |

|  | **CENTAURO-2** | |
| --- | --- | --- |
|  | **Group Ag+Al** | |
| **Platelet-corrected serum VEGF (pg/10^6^)** | | |
|  | **Week 0** | **Week 13** |
| CH07 | 2.593 | 1.541 |
| CQ13 | 4.984 | 4.843 |
| CQ28 | 2.437 | 2.627 |
| CH37 | 1.770 | 1.110 |
| CH45 | 2.719 | 0.388 |
| JL48 | 5.223 | 4.769 |
| JL11 | 1.657 | 1.719 |
| CH10 | 3.504 | 2.058 |

|  | **CENTAURO** | |
| --- | --- | --- |
|  | **Group ⅛Ag+V** | |
| **Platelet-derived VEGF (pg/10^6^)** | | |
|  | **Week 0** | **Week 13** |
| CH01 | 2.140 | 0.596 |
| CH02 | 1.727 | 1.792 |
| CH03 | 1.217 | 3.201 |
| CH06 | 1.547 | 2.031 |
| CH07 | 0.836 | 1.015 |
| CH08 | 1.446 | 0.496 |
| CH09 | 3.394 | 6.925 |
| CH10 | 2.015 | 1.427 |

|  | **CENTAURO** | |
| --- | --- | --- |
|  | **Group ¼Ag+V** | |
| **Platelet-derived VEGF (pg/10^6^)** | | |
|  | **Week 0** | **Week 13** |
| CH11 | 0.693 | 0.627 |
| CH14 | 0.434 | 0.817 |
| CH15 | 4.187 | 0.865 |
| CH16 | 1.797 | 0.618 |
| CQ17 | 3.427 | 1.428 |
| CH25 | 0.894 | 0.980 |
| CH26 | 1.651 | 0.858 |
| CH27 | 1.286 | 2.122 |

|  | **CENTAURO** | |
| --- | --- | --- |
|  | **Group Ag+V** | |
| **Platelet-derived VEGF (pg/10^6^)** | | |
|  | **Week 0** | **Week 13** |
| CH18 | 3.969 | 1.986 |
| CH19 | 0.270 | 0.381 |
| CH21 | 3.715 | 2.026 |
| CH22 | 1.683 | 1.550 |
| CH23 | 2.095 | 1.795 |
| CH24 | 1.738 | 1.064 |
| CH29 | 0.779 | 0.260 |
| CH30 | 1.526 | 0.877 |

|  | **CENTAURO-2** | |
| --- | --- | --- |
|  | **Group Ag+V** | |
| **Platelet-derived VEGF (pg/10^6^)** | | |
|  | **Week 0** | **Week 13** |
| CQ03 | 2.861 | 1.108 |
| CH04 | 0.813 | 2.269 |
| CH17 | 1.872 | 0.624 |
| CH24 | 0.883 | 0.161 |
| CH32 | 3.371 | 0.883 |
| JL41 | 0.437 | 0.352 |
| JL42 | 2.976 | 1.267 |

|  | **CENTAURO-2** | |
| --- | --- | --- |
|  | **Group Ag+2V** | |
| **Platelet-derived VEGF (pg/10^6^)** | | |
|  | **Week 0** | **Week 13** |
| CH15 | 0.996 | 1.332 |
| CH19 | 1.446 | 0.208 |
| CH27 | 2.347 | 1.604 |
| CH39 | 1.485 | 1.381 |
| JL49 | 0.901 | 0.128 |
| JL22 | 1.610 | 1.467 |
| CQ44 | 1.558 | 4.765 |

|  | **CENTAURO-2** | |
| --- | --- | --- |
|  | **Group 2Ag+V** | |
| **Platelet-derived VEGF (pg/10^6^)** | | |
|  | **Week 0** | **Week 13** |
| CH16 | 1.857 | 0.675 |
| JL29 | 0.566 | 0.520 |
| JL30 | 9.359 | 2.952 |
| CH33 | 3.190 | 0.681 |
| JL43 | 0.166 | 0.294 |
| CH50 | 3.022 | 0.345 |
| JL12 | 2.507 | 0.388 |
| CH35 | 1.754 | 0.975 |

|  | **CENTAURO-2** | |
| --- | --- | --- |
|  | **Group ½Ag+Al** | |
| **Platelet-derived VEGF (pg/10^6^)** | | |
|  | **Week 0** | **Week 13** |
| CH08 | 2.086 | 0.968 |
| CH09 | 2.812 | 5.453 |
| CH20 | 4.166 | 3.711 |
| CH25 | 1.416 | 0.951 |
| CH46 | 1.893 | 1.603 |
| JL47 | 1.556 | 2.198 |
| CH18 | 4.845 | 4.778 |
| JL23 | 3.758 | 3.765 |

|  | **CENTAURO-2** | |
| --- | --- | --- |
|  | **Group Ag+Al** | |
| **Platelet-derived VEGF (pg/10^6^)** | | |
|  | **Week 0** | **Week 13** |
| CH07 | 1.214 | 0.735 |
| CQ13 | 4.382 | 3.458 |
| CQ28 | 1.731 | 1.173 |
| CH37 | 1.046 | 0.554 |
| CH45 | 2.465 | 0.266 |
| JL48 | 4.676 | 4.224 |
| JL11 | 1.538 | 1.492 |
| CH10 | 2.531 | 1.361 |

|  | **CENTAURO** | |
| --- | --- | --- |
|  | **Group ⅛Ag+V** | |
| **Platelet VEGF** | | |
|  | **Week 0** | **Week 13** |
| CH01 | 1.329 | 0.372 |
| CH02 | 1.121 | 1.193 |
| CH03 | 0.861 | 2.138 |
| CH06 | 0.918 | 1.322 |
| CH07 | 0.511 | 0.622 |
| CH08 | 0.776 | 0.279 |
| CH09 | 2.233 | 4.418 |
| CH10 | 1.250 | 0.939 |

|  | **CENTAURO** | |
| --- | --- | --- |
|  | **Group ¼Ag+V** | |
| **Platelet VEGF** | | |
|  | **Week 0** | **Week 13** |
| CH11 | 0.407 | 0.386 |
| CH14 | 0.252 | 0.473 |
| CH15 | 2.424 | 0.545 |
| CH16 | 1.123 | 0.440 |
| CQ17 | 1.991 | 0.841 |
| CH25 | 0.510 | 0.560 |
| CH26 | 1.148 | 0.526 |
| CH27 | 0.777 | 1.375 |

|  | **CENTAURO** | |
| --- | --- | --- |
|  | **Group Ag+V** | |
| **Platelet VEGF** | | |
|  | **Week 0** | **Week 13** |
| CH18 | 2.473 | 1.182 |
| CH19 | 0.175 | 0.239 |
| CH21 | 2.147 | 1.252 |
| CH22 | 1.157 | 0.829 |
| CH23 | 1.460 | 1.278 |
| CH24 | 1.003 | 0.597 |
| CH29 | 0.507 | 0.151 |
| CH30 | 0.920 | 0.503 |

|  | **CENTAURO-2** | |
| --- | --- | --- |
|  | **Group Ag+V** | |
| **Platelet VEGF (pg/10^6^)** | | |
|  | **Week 0** | **Week 13** |
| CQ03 | 1.91 | 0.73 |
| CH04 | 0.46 | 1.43 |
| CH17 | 1.07 | 0.39 |
| CH24 | 0.51 | 0.10 |
| CH32 | 2.20 | 0.55 |
| JL41 | 0.30 | 0.24 |
| JL42 | 2.01 | 0.80 |

|  | **CENTAURO-2** | |
| --- | --- | --- |
|  | **Group Ag+2V** | |
| **Platelet VEGF (pg/10^6^)** | | |
|  | **Week 0** | **Week 13** |
| CH15 | 0.62 | 0.882 |
| CH19 | 0.92 | 0.130 |
| CH27 | 1.46 | 1.000 |
| CH39 | 0.91 | 0.820 |
| JL49 | 0.55 | 0.080 |
| JL22 | 0.93 | 0.877 |
| CQ44 | 1.09 | 3.290 |

|  | **CENTAURO-2** | |
| --- | --- | --- |
|  | **Group 2Ag+V** | |
| **Platelet VEGF (pg/10^6^)** | | |
|  | **Week 0** | **Week 13** |
| CH16 | 1.25 | 0.43 |
| JL29 | 0.35 | 0.32 |
| JL30 | 8.19 | 2.05 |
| CH33 | 1.91 | 0.39 |
| JL43 | 0.11 | 0.23 |
| CH50 | 1.94 | 0.22 |
| JL12 | 1.66 | 0.25 |
| CH35 | 1.13 | 0.69 |

|  | **CENTAURO-2** | |
| --- | --- | --- |
|  | **Group ½Ag+Al** | |
| **Platelet VEGF (pg/10^6^)** | | |
|  | **Week 0** | **Week 13** |
| CH08 | 1.46 | 0.660 |
| CH09 | 1.76 | 3.530 |
| CH20 | 2.65 | 2.461 |
| CH25 | 0.90 | 0.630 |
| CH46 | 1.07 | 0.960 |
| JL47 | 1.11 | 1.600 |
| CH18 | 3.64 | 3.540 |
| JL23 | 2.59 | 2.458 |

|  | **CENTAURO-2** | |
| --- | --- | --- |
|  | **Group Ag+Al** | |
| **Platelet VEGF (pg/10^6^)** | | |
|  | **Week 0** | **Week 13** |
| CH07 | 0.79 | 0.47 |
| CQ13 | 2.57 | 1.87 |
| CQ28 | 1.17 | 0.78 |
| CH37 | 0.72 | 0.36 |
| CH45 | 1.44 | 0.15 |
| JL48 | 3.19 | 2.91 |
| JL11 | 1.01 | 1.03 |
| CH10 | 1.75 | 0.88 |

|  | **CENTAURO** | |
| --- | --- | --- |
|  | **Group ⅛Ag+V** | |
| **Platelet (10^9^/L)** | | |
|  | **Week 0** | **Week 13** |
| CH01 | 238. | 239. |
| CH02 | 264. | 352. |
| CH03 | 234. | 175. |
| CH06 | 172. | 244. |
| CH07 | 215. | 225. |
| CH08 | 207. | 270. |
| CH09 | 265. | 317. |
| CH10 | 235. | 257. |

|  | **CENTAURO** | |
| --- | --- | --- |
|  | **Group ¼Ag+V** | |
| **Platelet (10^9^/L)** | | |
|  | **Week 0** | **Week 13** |
| CH11 | 329. | 323. |
| CH14 | 257. | 381. |
| CH15 | 194. | 246. |
| CH16 | 335. | 389. |
| CQ17 | 199. | 204. |
| CH25 | 252. | 223. |
| CH26 | 356. | 521. |
| CH27 | 358. | 544. |

|  | **CENTAURO** | |
| --- | --- | --- |
|  | **Group Ag+V** | |
| **Platelet (10^9^/L)** | | |
|  | **Week 0** | **Week 13** |
| CH18 | 133. | 176. |
| CH19 | 244. | 238. |
| CH21 | 309. | 390. |
| CH22 | 226. | 183. |
| CH23 | 263. | 285. |
| CH24 | 323. | 379. |
| CH29 | 344. | 346. |
| CH30 | 187. | 275. |

|  | **CENTAURO-2** | |
| --- | --- | --- |
|  | **Group Ag+V** | |
| **Platelet (10^9^/L)** | | |
|  | **Week 0** | **Week 13** |
| CQ03 | 194. | 227. |
| CH04 | 506. | 375. |
| CH17 | 293. | 299. |
| CH24 | 162. | 152. |
| CH32 | 225. | 245. |
| JL41 | 139. | 188. |
| JL42 | 297. | 259. |

|  | **CENTAURO-2** | |
| --- | --- | --- |
|  | **Group Ag+2V** | |
| **Platelet (10^9^/L)** | | |
|  | **Week 0** | **Week 13** |
| CH15 | 261. | 225. |
| CH19 | 185. | 155. |
| CH27 | 141. | 206. |
| CH39 | 299. | 350. |
| JL49 | 155. | 154. |
| JL22 | 284. | 264. |
| CQ44 | 327. | 567. |

|  | **CENTAURO-2** | |
| --- | --- | --- |
|  | **Group 2Ag+V** | |
| **Platelet (10^9^/L)** | | |
|  | **Week 0** | **Week 13** |
| CH16 | 239. | 299. |
| JL29 | 470. | 432. |
| JL30 | 106. | 328. |
| CH33 | 284. | 251. |
| JL43 | 459. | 663. |
| CH50 | 318. | 367. |
| JL12 | 236. | 218. |
| CH35 | 255. | 298. |

|  | **CENTAURO-2** | |
| --- | --- | --- |
|  | **Group ½Ag+Al** | |
| **Platelet (10^9^/L)** | | |
|  | **Week 0** | **Week 13** |
| CH08 | 161. | 201. |
| CH09 | 105. | 96. |
| CH20 | 344. | 395. |
| CH25 | 317. | 223. |
| CH46 | 219. | 201. |
| JL47 | 528. | 457. |
| CH18 | 299. | 365. |
| JL23 | 381. | 477. |

|  | **CENTAURO-2** | |
| --- | --- | --- |
|  | **Group Ag+Al** | |
| **Platelet (10^9^/L)** | | |
|  | **Week 0** | **Week 13** |
| CH07 | 229. | 285. |
| CQ13 | 162. | 147. |
| CQ28 | 194. | 274. |
| CH37 | 359. | 248. |
| CH45 | 221. | 224. |
| JL48 | 287. | 237. |
| JL11 | 226. | 223. |
| CH10 | 345. | 442. |

|  | **CENTAURO** | |
| --- | --- | --- |
|  | **Group ⅛Ag+V** | |
| **Hematocrit** | | |
|  | **Week 0** | **Week 13** |
| CH01 | 0.379 | 0.376 |
| CH02 | 0.351 | 0.334 |
| CH03 | 0.292 | 0.332 |
| CH06 | 0.407 | 0.349 |
| CH07 | 0.389 | 0.387 |
| CH08 | 0.463 | 0.438 |
| CH09 | 0.342 | 0.362 |
| CH10 | 0.380 | 0.342 |

|  | **CENTAURO** | |
| --- | --- | --- |
|  | **Group ¼Ag+V** | |
| **Hematocrit** | | |
|  | **Week 0** | **Week 13** |
| CH11 | 0.413 | 0.384 |
| CH14 | 0.419 | 0.421 |
| CH15 | 0.421 | 0.370 |
| CH16 | 0.375 | 0.288 |
| CQ17 | 0.419 | 0.411 |
| CH25 | 0.419 | 0.428 |
| CH26 | 0.305 | 0.387 |
| CH27 | 0.396 | 0.352 |

|  | **CENTAURO** | |
| --- | --- | --- |
|  | **Group Ag+V** | |
| **Hematocrit** | | |
|  | **Week 0** | **Week 13** |
| CH18 | 0.377 | 0.405 |
| CH19 | 0.350 | 0.374 |
| CH21 | 0.422 | 0.382 |
| CH22 | 0.313 | 0.465 |
| CH23 | 0.303 | 0.288 |
| CH24 | 0.423 | 0.439 |
| CH29 | 0.349 | 0.420 |
| CH30 | 0.397 | 0.426 |

|  | **CENTAURO-2** | |
| --- | --- | --- |
|  | **Group Ag+V** | |
| **Hematocrit** | | |
|  | **Week 0** | **Week 13** |
| CQ03 | 0.331 | 0.339 |
| CH04 | 0.432 | 0.370 |
| CH17 | 0.430 | 0.374 |
| CH24 | 0.422 | 0.395 |
| CH32 | 0.348 | 0.375 |
| JL41 | 0.317 | 0.325 |
| JL42 | 0.326 | 0.367 |

|  | **CENTAURO-2** | |
| --- | --- | --- |
|  | **Group Ag+2V** | |
| **Hematocrit** | | |
|  | **Week 0** | **Week 13** |
| CH15 | 0.375 | 0.338 |
| CH19 | 0.362 | 0.363 |
| CH27 | 0.379 | 0.374 |
| CH39 | 0.388 | 0.407 |
| JL49 | 0.385 | 0.395 |
| JL22 | 0.423 | 0.402 |
| CQ44 | 0.300 | 0.310 |

|  | **CENTAURO-2** | |
| --- | --- | --- |
|  | **Group 2Ag+V** | |
| **Hematocrit** | | |
|  | **Week 0** | **Week 13** |
| CH16 | 0.326 | 0.374 |
| JL29 | 0.382 | 0.377 |
| JL30 | 0.125 | 0.304 |
| CH33 | 0.400 | 0.429 |
| JL43 | 0.313 | 0.209 |
| CH50 | 0.358 | 0.352 |
| JL12 | 0.338 | 0.360 |
| CH35 | 0.308 | 0.290 |

|  | **CENTAURO-2** | |
| --- | --- | --- |
|  | **Group ½Ag+Al** | |
| **Hematocrit** | | |
|  | **Week 0** | **Week 13** |
| CH08 | 0.298 | 0.316 |
| CH09 | 0.375 | 0.353 |
| CH20 | 0.363 | 0.337 |
| CH25 | 0.364 | 0.340 |
| CH46 | 0.433 | 0.401 |
| JL47 | 0.288 | 0.271 |
| CH18 | 0.248 | 0.260 |
| JL23 | 0.310 | 0.347 |

|  | **CENTAURO-2** | |
| --- | --- | --- |
|  | **Group Ag+Al** | |
| **Hematocrit** | | |
|  | **Week 0** | **Week 13** |
| CH07 | 0.351 | 0.357 |
| CQ13 | 0.414 | 0.460 |
| CQ28 | 0.325 | 0.339 |
| CH37 | 0.308 | 0.352 |
| CH45 | 0.414 | 0.430 |
| JL48 | 0.317 | 0.312 |
| JL11 | 0.342 | 0.310 |
| CH10 | 0.309 | 0.356 |
